# Effects of tDCS combined with TENS in relieving pain and improving gait patterns during stepping over obstacles among older adults with knee osteoarthritis

**DOI:** 10.3389/fspor.2025.1631357

**Published:** 2025-09-17

**Authors:** Xinmeng Zhang, Dongmei Wang, Qingqing Song, Xin Luo, Yubin Ge, Peixin Shen, Qipeng Song

**Affiliations:** ^1^ Graduate School, Shandong Sport University, Jinan, China; ^2^ Biomechanics Laboratory College of Human Movement Science, Beijing Sport University, Beijing, China; ^3^ College of Sports and Health, Shandong Sport University, Jinan, China

**Keywords:** knee pain, osteoarthritis, transcranial direct current stimulation, transcutaneous electrical nerve stimulation, obstacle crossing

## Abstract

**Purpose:**

Older adults with knee osteoarthritis (KOA) exhibit an elevated risk of falls during obstacle negotiation, primarily due to pain-induced gait deviations. While transcutaneous electrical nerve stimulation (TENS) offers modest pain relief and limited gait modulation, combining it with transcranial direct current stimulation (tDCS) may enhance the effects. This study evaluated the comparative efficacy of tDCS + TENS vs. TENS alone in mitigating pain and optimizing gait patterns during obstacle crossing in older adults with KOA.

**Methods:**

Twenty-three participants with KOA (mean age: 67.6 ± 5.0 years; BMI: 25.9 ± 2.4 kg/m^2^) were randomized to either tDCS + TENS (*n* = 12; 7F/5M) or TENS-only (*n* = 11; 7F/4M) groups. Both interventions involved 20-minute sessions, administered thrice weekly for six weeks. Outcome measures included pain intensity (visual analog scale, VAS) and gait variables (foot clearance height, crossing velocity) assessed pre- (week 0) and post-intervention (week 7). Data were analyzed using mixed-design two-way ANOVAs with Bonferroni corrections.

**Results:**

Statistically significant group-by-time interactions were observed for pain (*p* = 0.002, *η*^2^_p_ = 0.378), foot clearance (*p* = 0.038, *η*^2^_p_ = 0.190), and crossing velocity (*p* < 0.001, *η*^2^_p_ = 0.588). *post hoc* analyses revealed that the tDCS + TENS group (week0 = 4.72 ± 1.01, week7 = 1.98 ± 0.88, *p* < 0.001) experienced significantly greater reductions in pain scores compared to the TENS-only group (week0 = 5.02 ± 1.19, week7 = 3.56 ± 1.18, *p* < 0.001); tDCS + TENS group experienced significantly greater improvements in foot clearance (week0 = 0.19 ± 0.04, week7 = 0.20 ± 0.03, *p* < 0.001) and crossing velocity (week0 = 0.53 ± 0.11, week7 = 0.62 ± 0.08, *p* < 0.001), compared to the TENS-only group (week0 = 0.17 ± 0.02, week7 = 0.17 ± 0.02, *p* < 0.001; week0 = 0.52 ± 0.09, week7 = 0.54 ± 0.09).

**Conclusion:**

The combination of tDCS and TENS significantly outperformed TENS-only in reducing pain and enhancing gait adaptability during obstacle negotiation in older adults with KOA. These findings support the integration of tDCS as an adjunctive neuromodulatory strategy to amplify the therapeutic benefits of TENS in this population.

## 1 Introduction

Knee osteoarthritis (KOA) is a chronic degenerative joint disorder characterized by progressive damage to articular cartilage, subchondral bone, and the synovial membrane (1). It ranks among the top five causes of disability in older adults (2), with a global prevalence exceeding 645 million individuals (3).

Obstacle negotiation presents distinct challenges for older adults with KOA, as this task exacerbates pain and elevates fall risk. Stepping over obstacles elicits greater pain intensity compared to level walking (4), a hallmark symptom of KOA (5). Approximately 50% of falls in this population occur during obstacle crossing (6), frequently resulting in fractures or mortality (7). Foot clearance, defined as the vertical distance between the foot and the obstacle during the swing phase of gait, is critical for fall avoidance, as most trips occur due to inadvertent contact between the swinging limb and the obstacle. Reduced foot clearance and stepping height is strongly associated with tripping and falls (8). Additionally, patients with KOA exhibit longer single-leg support time and slower gait speeds (9), with a 0.1 m/s decrease in velocity linked to a 10% decline in physical performance capacity (10). These gait alterations may arise from decreased lower limb flexion angles in the leading leg, reduced vertical impulse from the trailing leg during swing initiation, or insufficient propulsive force to maintain gait speed (11, 12).

Standard KOA interventions include pharmacological therapy, surgical procedures, and physical agent modalities (13). Pharmacological analgesics provide transient pain relief but are associated with gastrointestinal and cardiovascular adverse effects (14), while surgical options may be contraindicated in older adults due to comorbidities (15). Physical therapies are favored for their rapid efficacy and safety profiles (16), with transcutaneous electrical nerve stimulation (TENS) being a widely recommended physiotherapy (17). TENS delivers electrical currents via cutaneous electrodes to modulate peripheral pain pathways by activating large-diameter Aβ afferent fibers, which enhance inhibitory interneuronal activity in the dorsal horn of the spinal cord (18). However, TENS exhibits limited analgesic duration and modest effects on functional outcomes (19).

The limited analgesic and functional efficacy of TENS in KOA may be attributed to its exclusively peripheral mechanism of action. According to the classic gate control theory, conventional peripheral physiotherapy (e.g., TENS) modulates pain by activating large-diameter Aβ afferent fibers, which enhance inhibitory interneuronal activity within the dorsal horn of the spinal cord, thereby “closing the gate” to nociceptive input (20). However, the gate control theory also posits a central inhibitory pathway. Aβ fiber signals are rapidly transmitted to the brainstem and cortex, where descending projections modulate spinal gate dynamics via the periaqueductal gray and rostral ventromedial medulla (20). While TENS targets peripheral nociceptive pathways, it fails to directly address central sensitization, a hallmark of chronic KOA pain and disability (21).

Central sensitization in KOA arises from sustained nociceptive input driven by synovial inflammation and sterile inflammation of local soft tissues, which activates peripheral nociceptors and triggers increased neurotransmitter release at primary afferent terminals in the spinal dorsal horn (22). This persistent afferent barrage enhances the responsiveness of nociceptive neurons, leading to heightened pain sensitivity and exaggerated responses to mild stimuli, perpetuating chronic pain (22). Additionally, central sensitization may contribute to functional impairments, including altered gait patterns that further elevate fall risk (23).

Transcranial direct current stimulation (tDCS) is a non-invasive brain stimulation technique that delivers low-intensity direct current via surface electrodes (anode and cathode) positioned over targeted cortical regions (24). By inducing subthreshold shifts in neuronal membrane polarization, tDCS modulates cortical excitability: anodal stimulation enhances excitability, while cathodal stimulation suppresses it. This neuroplastic modulation may attenuate central sensitization-related pain hypersensitivity by activating descending inhibitory pathways in the spinal dorsal horn (25).

Overall, TENS provides transient pain relief via peripheral mechanisms but has limited effects on functional outcomes (19). In contrast, tDCS modulates cortical excitability and central pain processing, potentially improving gait patterns through enhanced neural plasticity (25, 26). A combined tDCS + TENS intervention may synergistically address pain and gait deficits in older adults with KOA by integrating peripheral analgesia with central neuroplasticity enhancement. However, no prior studies have evaluated this approach. Therefore, this study aimed to investigate the effects of a 6-week tDCS combined TENS intervention on pain relief and obstacle-crossing gait improvement (including foot clearance and crossing velocity) among older adults with KOA, compared to TENS alone. We hypothesize that (1) both TENS + tDCS and TENS alone will reduce pain scores and improve obstacle-crossing gait patterns (i.e., increased foot clearance and crossing velocity) in older adults with KOA, and (2) TENS + tDCS intervention will demonstrate superior efficacy compared to TENS alone in reducing pain and enhancing gait adaptability.

## 2 Materials and methods

### 2.1 Sample size estimate

An *a priori* power analysis conducted by the G*Power 3.1 software (University of Düsseldorf, Düsseldorf, Germany) indicated that a minimum of 22 participants should be recruited to obtain an alpha level of 0.05 and a statistical power of 0.95 based on a previous study: pain scores decreased more significantly in KOA patients who received 4-week of TENS combined with tDCS interventions compared to those who received only 4-week of TENS combined with sham tDCS interventions with a significant group-by-intervention interaction (*p* = 0.038, *η*^2^_p_ = 0.101) detected in the pain scores using a mixed design two-way ANOVA (27).

### 2.2 Participants

All participants were recruited from local communities via flyer distribution and presentations from Sep 2024 to Jan 2025. Fifty individuals were screened for eligibility based on the following inclusion criteria: (a) aged 65 years or older; (b) diagnosed with unilateral or bilateral KOA per the American College of Rheumatology clinical criteria (28); (c) Kellgren/Lawrence radiographic grade 2 or 3. Exclusion criteria included: (a) neurological or neuromuscular disorders affecting the knee (other than KOA); (b) history of lower extremity joint surgery or fractures within the past 3 months; (c) planned total knee replacement in the coming months; (d) chronic, disabling back, hip, ankle, or foot pain interfering with daily activities; (e) severe cognitive impairment (Mini-Mental State Examination score <24); (f) intolerance to electrical stimulation (e.g., pacemaker implantation, unusual pinprick sensation).

**Figure 1:**
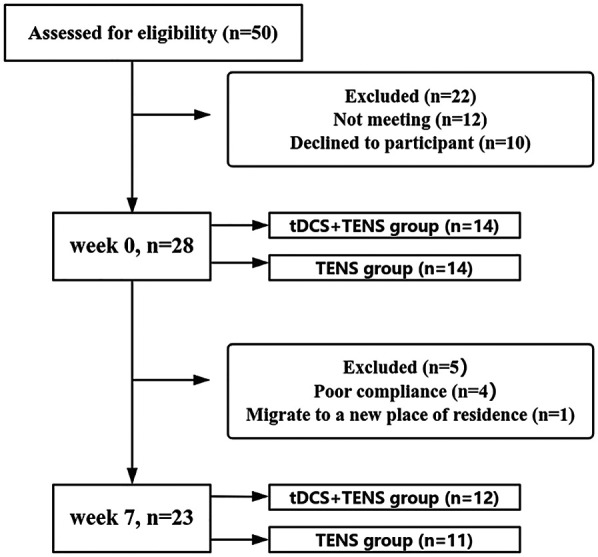
Participant flow chart. Participation flow chart from week0 to week7. The final analysis included data from 23 participants. Twenty-seven participants were excluded from the original 50 recruited due to various reasons. tDCS, transcranial direct current stimulation; TENS, transcutaneous electrical nerve stimulation.

Twenty-eight eligible participants were randomly allocated (1:1 ratio) to tDCS + TENS or TENS groups using sequentially numbered, opaque, sealed envelopes containing group assignments. The tDCS + TENS group received active tDCS combined with TENS, while the TENS group received sham tDCS combined with TENS, over 6 weeks (three 20-minute sessions weekly). Five participants dropped out by week_7_, one for relocation and four for poor compliance. Final analysis included 23 participants (12 in the tDCS + TENS group and 11 in the TENS group) (Figure 1). All participants provided written informed consent. The study was approved by the Ethics Committee of Exercise Science, Shandong Sport University (20233037), adhering to the Declaration of Helsinki.

### 2.3 Transcranial direct current stimulation (tDCS) intervention

**Figure 2:**
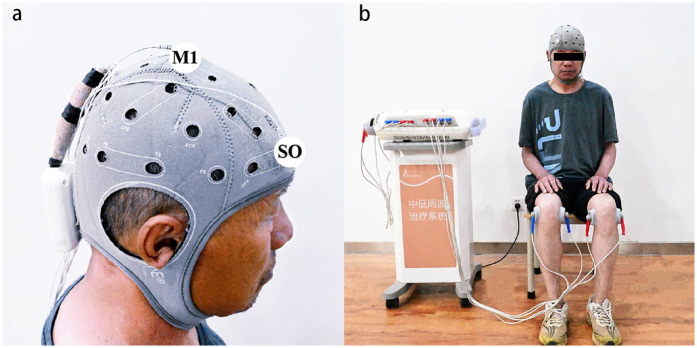
Illustration of tDCS electrode and TENS surface electrode placement. **(a)** The illustration of the tDCS electrode placement. The anode electrode was placed over the M1 on the contralateral primary motor cortex (M1) of the affected knee, the cathode electrode was placed over supraorbital (SO) area. **(b)** The illustration of the TENS electrode placement. Two surface electrodes were placed opposite each other on the medial and lateral sides of the knee joints. tDCS, transcranial direct current stimulation; TENS, transcutaneous electrical nerve stimulation.

A tDCS device (Starstim8, Neuroelectronics, Spain) delivered stimulation via two 5 cm diameter rubberized circular electrodes. The anode was precisely positioned over the primary motor cortex (M1) at the Cz electrode site of the 10–20 EEG system. Cz is located at the skull midline, midway between the nasion (nasal root) and inion (external occipital protuberance), corresponding to the lower limb motor cortex. The cathode was placed over the ipsilateral supraorbital (SO) area (FP2 or FP1 of the non-dominant hemisphere), targeting the hemisphere contralateral to the affected knee (determined by higher Kellgren-Lawrence grade or self-reported pain intensity). This formed the M1-SO montage (27) (Figure 2a). Active tDCS delivered a constant current of 2 mA, ramped from 0 mA to 2 mA over 30 s, maintained at 2 mA for 19 min, and tapered to 0 mA over 30 s (total session duration: 20 min). Sham stimulation mirrored electrode placement and initial ramp-up (30 s at 2 mA), followed by immediate shutdown to mimic sensory effects.

### 2.4 Transcutaneous electrical nerve stimulation (TENS) intervention

Stimulation was delivered via the Low and Medium Frequency Therapy System (Junde Medical Equipment Co., Ltd., Model IN-1300, Hebei, China) using TENS modalities, at the same time when participants received tDCS intervention. Two circular surface electrodes (diameter: 5 cm) were positioned on the medial and lateral sides of the knees, approximately 5 cm apart and centered on the pain site (Figure 2b). Conventional TENS parameters included: 100 Hz frequency, 100 μs pulse width, and a balanced biphasic square waveform. The intensity range of the TENS device is fixed at 0–35 mA and can be continuously adjusted within this range. In this study, the intensities received by the participants were mostly concentrated in the range of 15–25 mA.

### 2.5 Stepping-over obstacle test

**Figure 3:**
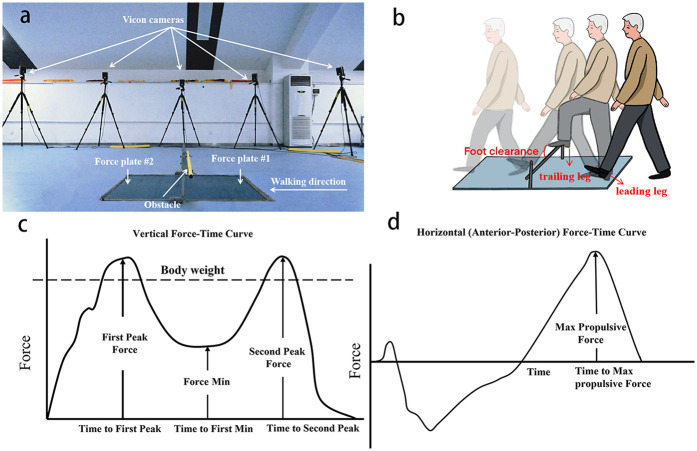
Diagram of the obstacle-crossing setup and variables. **(a)** Obstacle and the force platforms setup. **(b)** Diagram of stepping over the obstacle. **(c)** Vertical ground reaction force-time curve. **(d)** Horizontal (anterior–posterior) ground reaction force–time curves.

Each participant walked at a self-selected pace on an 8-m walkway and stepped over an obstacle with a height of 20% of each participant's leg length (29). Two force platforms (90*60*10 cm, AMTI, BP600900, USA) were placed adjacent with the long edges and on either side of the obstacle (Figure 3a). The trailing leg steps on the near side of the force platform first, and then the leading leg steps on the far side of the force platform on the other side of the obstacle. Before the tests, the participants were asked to familiarize themselves with the obstacle-stepping process. Forty-three markers were placed on bony landmarks according to the protocol 13-segment whole body model. Three-dimensional kinematics data were collected by a twelve-camera motion analysis system (Vicon, Oxford Metrics Ltd., UK) at 100 Hz. The kinematic data were internally synchronized with the ground reaction force data collected using the force platforms at 1,000 Hz. Each participant was instructed to step over the obstacle using their affected leg (Figure 3b). Three successful trials were collected, a successful trial was defined as a trial the participants used the affected leg as the leading leg and had no contact with the obstacles, and no gait adjustments were adopted during the process.

### 2.6 Pain scores

Visual analog scale (VAS) was used to assess patient's pain (30). It consists of a 10-cm horizontal line with endpoints labeled “0” (no pain) and “10” (worst possible pain). Participants were instructed to mark their pain level on the line immediately after crossing the obstacle at weeks 0 and 7. Higher scores indicate greater pain intensity.

### 2.7 Variables

Foot clearance was determined as vertical height between the lowest point on the leading foot and obstacle when the foot was directly over the obstacle (29). Crossing velocity was calculated as the mean anterior-posterior velocity of the center of mass during the stepping-over stride cycle, beginning at the trailing leg heel-strike on the force platform, ending at the next heel-strike of the same leg (31). Hip and knee flexion and ankle dorsiflexion angles were measured as joint Euler angles when the toe marker of the leading leg was directly above the obstacle.

Vertical impulse was computed by integrating the vertical ground reaction force (vGRF)-time curve of the trailing limb from trailing limb heel contact (detected when vGRF >20 N) to trailing limb toe-off (vGRF <20 N). Propulsive impulse was computed by integrating the anterior-posterior ground reaction force (AP GRF)-time curve of the trailing limb during the propulsion phase, defined as the period from the transition to positive AP GRF (forward-directed force) to trailing limb toe-off (vRF <20 N). Stepping height was defined as the maximum vertical distance between the heel marker of the leading limb and the ground surface during the swing phase. Support time was defined as the duration from heel strike of the trailing limb to toe-off of the same limb during obstacle negotiation, measured using force platform data (vertical ground reaction force >20 N threshold).

### 2.8 Data reduction

Helen Hayes Model in Visual-3D software (C-motion, Germantown, MD) was uesed to process with data. Joint angles were computed via Euler rotations, and hip, knee, and ankle joint centers were determined from marker positions and participant-specific anatomical measurements. Vertical impulse was calculated as the time-integrated vertical GRF during stance phase (Figure 3c), whereas propulsive impulse represented the time integrated anterior-posterior GRF component. GRF were sampled at 1,000 Hz, normalized by body weight (BW) to enable inter-subject comparisons and expressed as a percentage of stance phase duration (Figure 3d). The kinematic and kinetic data were filtered using a fourth-order low-pass Butterworth filter with cutoff frequencies of 6 and 50 Hz (32). Vertical and propulsive impulse was derived from normalized force-time data using trapezoidal-rule integration.

### 2.9 Statistics

The normality of data was verified using Shapiro–Wilk tests. Mixed-design two-way ANOVAs were used to verify the main effects of group (tDCS + TENS vs. TENS) and time (week0 vs. week7), and their interactions. If a significant interaction was detected, Bonferroni adjusted post hocs would be conducted. Partial eta square(*η*^2^_p_) was used to represent the effect size of main effects and interactions. The thresholds for *η*^2^_p_ were as follows: 0.01–0.06, small; 0.06–0.14, moderate; >0.14, large (33). Cohen's *d* was used to represent the effect size of the *post hoc* comparisons. The thresholds for *d* were as follows: <0.20, trivial; 0.21–0.50, small; 0.51–0.80, medium; >0.81, large (34). The significance level is set to 0.05, and *p*-value less than the level indicates a statistically significant result, meaning the observed data provide strong evidence against the null hypothesis.

## 3 Results

All dependent variables exhibited normal distribution, as verified through Shapiro–Wilk tests (*p* > 0.05). Chi-square tests revealed no statistically significant differences in sex (*p* = 0.795) and the side of the more affected leg (*p* = 0.795) between the two groups. Independent *t*-tests indicated no statistically significant differences in age (*p* = 0.828), height (*p* = 0.196), body mass (*p* = 0.055), and body mass index (*p* = 0.078) between the groups (Table 1).

**Table 1:** Baseline characteristics.

Group	tDCS + TENS group (*n* = 12)	TENS group (*n* = 11)	*p*
Sex	F (7, 58.3%), M (5, 41.7%)	F (7, 63.6%), M (4, 36.4%)	0.795
Affected leg	R (7, 58.3%), L (5, 41.7%)	R (7, 63.6%), L (4, 36.4%)	0.795
Age (y)	67.7 ± 5.0	67.5 ± 5.1	0.828
Height (cm)	159.2 ± 6.8	163.5 ± 10.0	0.196
Body mass (kg)	66.4 ± 6.8	69.1 ± 12.6	0.055
BMI (kg/m^2^)	26.2 ± 1.7	25.7 ± 2.8	0.078

Data were presented as mean ± standard deviation. Chi-square tests were used to compare differences in sex, and side of the affected leg. Independent *t*-tests were used to compare differences in age, height, body mass and BMI between the tDCS + TENS and TENS groups.

F, female; M, male; R, right; L, left; tDCS, transcranial direct current stimulation; TENS, transcutaneous electrical nerve stimulation.

### 3.1 Primary outcomes

**Figure 4:**
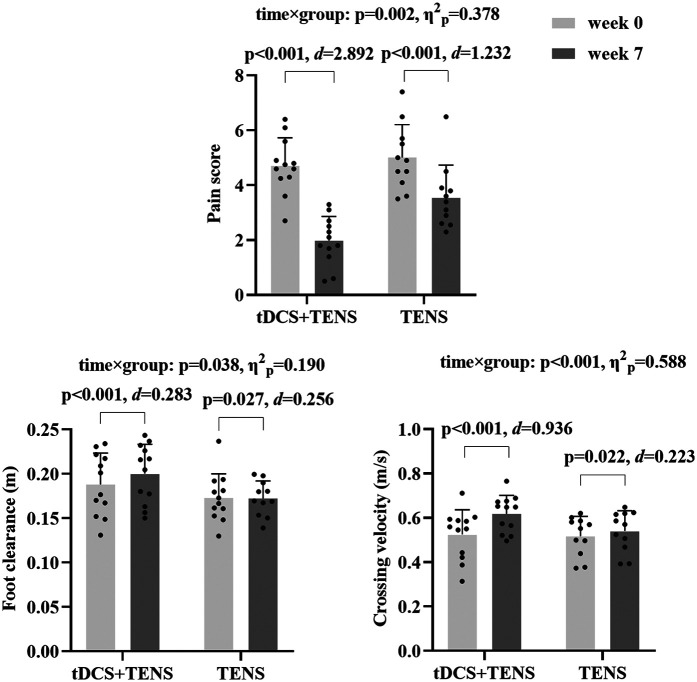
Primary outcomes. tDCS, transcranial direct current stimulation; TENS, transcutaneous electrical nerve stimulation.

As shown in Figure 4, significant time × group interactions were detected for pain score (*p* = 0.002, *η*^2^_p_ = 0.378), which decreased in both groups from week_0_ to week_7_ (tDCS + TENS: *p* < 0.001, *d* = 2.892; TENS: *p* < 0.001, *d* = 1.232), with greater reductions observed in the tDCS + TENS group compared to the TENS group. Significant time × group interactions were observed in crossing velocity (*p* < 0.001, *η*^2^_p_ = 0.588) and foot clearance (*p* = 0.038, *η*^2^_p_ = 0.190). Crossing velocity (tDCS + TENS: *p* < 0.001, *d* = 0.936; TENS: *p* = 0.022, *d* = 0.223) and foot clearance (tDCS + TENS: *p* < 0.001, *d* = 0.283; TENS: *p* = 0.027 *d* = 0.256) increased in both groups from week_0_ to week_7_, with greater improvements observed in the tDCS + TENS group compared to the TENS group.

**Figure 5:**
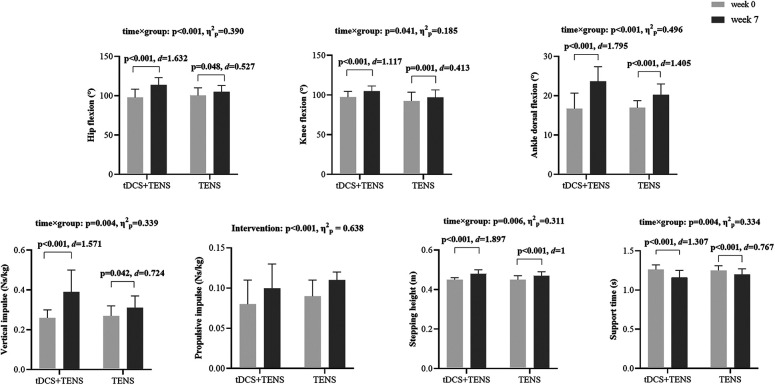
Secondary outcomes. tDCS, transcranial direct current stimulation; TENS, transcutaneous electrical nerve stimulation.

### 3.2 Secondary outcomes

As shown in Figure 5. Significant time × group interactions were detected in hip flexion (*p* < 0.001, *η*^2^_p_ = 0.390), knee flexion (*p* = 0.041, *η*^2^_p_ = 0.185), and ankle dorsiflexion (*p* < 0.001, *η*^2^_p_ = 0.496) angles. They increase in both groups from week_0_ to week_7_, with the tDCS + TENS group showing greater improvements than the TENS group (tDCS + TENS: hip flexion *p* < 0.001, *d* = 1.632; knee flexion *p* < 0.001, *d* = 1.117; ankle dorsiflexion *p* < 0.001, *d* = 1.795; TENS: hip flexion *p* = 0.048, *d* = 0.527; knee flexion *p* = 0.001, *d* = 0.413; ankle dorsiflexion *p* < 0.001, *d* = 1.405).

Significant time × group interactions were detected for vertical impulse (*p* = 0.004, *η*^2^_p_ = 0.339), which decreases in both groups from week_0_ to week_7_ (tDCS + TENS: *p* < 0.001, *d* = 1.571; TENS: *p* = 0.042, *d* = 0.724), and greater reductions in the tDCS + TENS group compared to the TENS group. Additionally, a significant main effect of intervention was detected for propulsive impulse in both groups (*p* < 0.001, *η*^2^_p_ = 0.638).

Significant time × group interactions were detected for sptepping height (*p* = 0.006, *η*^2^_p_ = 0.311) which increased in both groups from week0 to week7 (tDCS + TENS: *p* < 0.001, *d* = 1.897; TENS: *p* < 0.001, *d* = 1), with greater improvements observed in the tDCS + TENS group compared to the TENS group. Significant time × group interactions were detected for support time (*p* = 0.004, *η*^2^_p_ = 0.334) which increased in both groups from week0 to week7 (tDCS + TENS: *p* < 0.001, *d* = 1.307; TENS: *p* < 0.001, *d* = 0.767, with greater reductions observed in the tDCS + TENS group compared to the TENS group.

## 4 Discussion

The purpose of this study was to verify the effect of tDCS combined with TENS on pain and gait patterns during stepping over obstacle among older adults with KOA. These results supported hypotheses # 1 and 2, by pointing out that both interventions relieve pain and improving gait patterns during stepping over obstacles among older adults with KOA, while tDCS + TENS training has better effects.

Our study showed that both interventions were effective in reducing pain, while tDCS + TENS had better effects in older adults with KOA. The finding is supported by a previous study, which indicated that combination of tDCS and TENS is more effective in relieving pain for individuals with KOA than using TENS or tDCS only (35). According to the classic gate control theory, pain perception is regulated by a gating mechanism in the spinal cord. In the dorsal horn, central transmitting cells, known as T-cells (transmission cells), act as relays for nociceptive signals. Glial cells and inhibitory interneurons function as a “gate” by modulating input to T-cells. This gate is regulated by signals from large-diameter and small-diameter fibers, the former closes the gate while the later opens the gate. TENS works by activating large-fiber fibers, thereby enhancing the inhibitory effect of interneurons and closing the gate to block peripheral nociceptive input (20, 36). However, in chronic KOA, persistent peripheral inflammation may lead to central sensitization. tDCS, in contrast, promotes neural plasticity by modulating thalamocortical circuits and synaptic plasticity, which reduces abnormal neuronal discharges associated with central sensitization and alleviates chronic pain-related hyperalgesia (37). TENS reduces peripheral nociceptive input, while tDCS suppresses central pain amplification. Together, these interventions synergistically attenuate pain by targeting both peripheral and central mechanisms.

Our study demonstrated that both interventions were effective in increasing foot clearance during obstacle-stepping in older adults with KOA, while tDCS + TENS yielded superior effects to TENS alone, likely due to two factors. Firstly, adequate toe clearance relies on sufficient hip and knee flexion and ankle dorsiflexion (38); our secondary outcomes revealed that combined tDCS and TENS significantly increased these joint angles during obstacle-stepping in this population, supported by prior research showing both TENS and tDCS can improve joint range of motion during functional tasks (39). Secondly, adequate toe clearance may also stem from higher vertical impulse in the trailing leg and increased spanning height, as our secondary outcomes indicated that combined tDCS + TENS outperformed TENS alone in this regard. During obstacle-stepping, limb control depends on joint movement; TENS alleviates knee pain via spinal gating mechanisms (A*β*-fiber activation) and endogenous *β*-endorphin release, which reduces pain-induced inhibition of quadriceps and gastrocnemius activation (40). This improves knee flexion and ankle dorsiflexion during the swing phase while simultaneously strengthening push-off forces and increasing spanning height through enhanced muscle activation. The generated greater vertical impulse directly results in improved foot clearance during obstacle-stepping. tDCS effects target central neural networks to amplify motor control and propulsion efficiency. Anodal tDCS applied over the primary motor cortex (M1) contralateral to the affected knee enhances corticospinal tract excitability (41), increasing the firing rate of pyramidal neurons and improving the synchronization of motor unit recruitment in lower limb muscles. This heightened cortical drive optimizes joint movement control, enabling more precise regulation of hip, knee, and ankle angles, strengthening push-off forces and increasing spanning height, ultimately enhancing foot clearance. Together, these interventions synergistically amplifies both joint mobility and propulsion efficiency, thereby achieving greater foot clearance during obstacle-stepping. tDCS enhances neuronal firing rates by boosting excitability in intracortical and subcortical networks and promoting synaptic plasticity (42), thereby improving joint movement control and strengthening push-off forces, ultimately enhancing foot clearance. Together, these interventions synergistically amplifies both joint mobility and propulsion efficiency, thereby achieving greater foot clearance during obstacle-stepping.

Our study demonstrated both interventions were effective in increasing obstacle crossing velocity in older adults with KOA, while the tDCS combined TENS is more effective than TENS alone. The support time and time main effect of propulsive impulse from the secondary outcomes supported both tDCS + TENS and TENS increases crossing velocity. Firstly, tDCS improved motor unit synchronization in the plantarflexors, increasing the rate of force development during push-off (41). This allowed the support leg to generate sufficient propulsion in less time, shortening support duration without compromising stability. TENS reduced pain-related muscle inhibition, allowing the quadriceps and gluteals to better stabilize the knee and hip during support, reducing the need for prolonged ground contact (40). Sencondly, tDCS induces cortical depolarization in the primary motor cortex (M1), increasing corticospinal tract excitability (41). This neuromodulatory effect refines the coupling between cortical motor commands and peripheral muscle activation, minimizing delays between neural input and mechanical output. This effect improves motor unit synchronization of plantarflexor muscles, enhancing push-off force generation during terminal stance phase. TENS activates A*β*-fiber mediated spinal gating and promotes *β*-endorphin release. These mechanisms reduce in pain-related muscle inhibition directly enhance joint kinematics during propulsion (40). By relieving quadriceps inhibition, TENS enables fuller knee extension during late stance, a movement that increases the leverage of the support leg. This extended knee position shifts the body's COM forward relative to the support foot, amplifying the mechanical advantage for generating forward momentum (11). Simultaneously, reduced inhibition of plantarflexors promotes greater ankle plantarflexion during push-off, which augments the force of propulsion (12). However, the lack of time by group interaction in propulsive impulse indicated that there are other factors attribute to the superior effects of tDCS combined TENS intervention, which may be attribute to the two factors. Firstly, tDCS augments proprioceptive signal integration through anodal stimulation of the primary motor cortex (M1), which increases neuronal excitability in this region, thereby improving the processing of proprioceptive inputs from muscles and joints (e.g., signals from muscle spindles and Golgi tendon organs) (43). This enhanced integration refines real-time perception of limb position and movement dynamics, enabling patients to rapidly adjust foot trajectory and joint angles during stepping. By minimizing positional errors that typically induce deceleration or hesitation, the intervention optimizes movement efficiency, ultimately elevating overall crossing speed.Secondly, tDCS applied over the primary motor cortex (M1) enhances multi-joint coordination through synchronized activation of synergistic neural networks across the ankle, knee, and hip joints by modulating corticospinal excitability and thalamocortical connectivity (44). This optimization reduces kinematic delays during obstacle negotiation, such as inadequate toe clearance or excessive swing-leg flexion, leading to smoother gait transitions and increased overall speed.

This study has limitations. First, there was no follow-up after the 6-week intervention; it cannot be determined how long the effect of the intervention on relieving pain and improving gait patterns during stepping over obstacles in older adults with KOA lasted. Second, the obstacle height was set at only 20% of leg length; incorporating additional heights could enhance the generalizability of the findings. Third, this study included older adults with both unilateral and bilateral KOA, but the gait patterns for stepping over obstacles may differ. Additionally, this study highlights the temporal aspects of ground reaction forces during gait but doesn't explore how individual joints contribute to gait pattern differences. Future research should integrate kinematic and kinetic data to better understand biomechanical changes in KOA patients. Machine learning, which has been effective in identifying key gait features and subtle movement differences (45, 46), could be particularly useful. Using these techniques may help clarify each joint's role in gait variability, improving diagnostic accuracy and guiding targeted interventions.

## 5 Conclusion

Both the combination of tDCS and TENS and TENS-only were effective in relieving pain and improving gait patterns during obstacle crossing in older adults with KOA, while the combination of tDCS and TENS had superior efficacy. These findings support the integration of tDCS as an adjunctive neuromodulatory strategy to amplify the therapeutic benefits of TENS in this population.

## Data Availability

The original contributions presented in the study are included in the article/Supplementary Material, further inquiries can be directed to the corresponding author.
